# Supplementary material for: Antigen-Specific Antibody Signature Is Associated with COVID-19 Outcome
Source: Viruses. 2023 Apr 20;15(4):1018. doi: 10.3390/v15041018 (PMC10143282; doi:10.3390/v15041018)
Supplement: Supplementary file 1 [file viruses-15-01018-s001.zip › viruses-2142507-supplementary/viruses-2142507-Supplementary Materials/Salgado et.al_Supplementary Figures.pdf]

Supplementary material

Supplementary Figure 1: Nucleocapsid proteins and antigenicity

Codon optimized full length nucleocapsid (N) protein of SARS-CoV-2 (Gen Bank QHD43423.2, residues: 1–419) and its domains N1 (residues: 1–182), N2 (residues: 115–304) and N3 (residues: 245–419), containing a polyhistidine (6x-His) tag at the N terminus of each protein was expressed in Escherichia coli strain BL21 and purified by affinity and size-exclusion chromatography as previously described. Western blotting (WB) was used to test the antigenicity of all the recombinants proteins. (A) A total of 1 ⍰g of SARS-CoV full length N, N1, N2 and N3 proteins, with or without urea, were loaded onto 12% polyacrylamide gels and transferred to a nitrocellulose membrane using standard procedures. The membranes were blocked with 5% skimmed milk in phosphate buffer saline (PBS) for 1 hour at room temperature (RT). Blotted membranes were incubated for one hour at RT with 1:1000 dilution of primary antibodies. Next, RT-PCR positive COVID-19 patient plasma sample, pool of pre-pandemic COVID-19 negative plasma sample pool and anti-histidine tagged mouse monoclonal antibody (Sigma-Aldrich) with 5% milk in PBST (PBS + 0.05% Tween-20) was added to the membranes. After incubation, membranes were washed three times with PBST then incubated with goat anti-mouse IgG HRP-labelled (0.2⍰g/mL KPL, Seracare, USA) or goat anti-human IgG HRP-labelled (0.1⍰g/mL KPL, Seracare, USA) secondary antibody for one hour at RT. The membranes were again washed three times with PBST and the proteins were visualized using an enzymatic reaction by adding DAB (3,3',4,4' diaminobenzidine) (BioRad, EUA) and H2O2 mixture for 5 minutes. Reaction was blocked by washing membrane five-times with distilled water and membranes were dried overnight to be photographed.

Supplementary Figure 2: Humoral response among COVID-19 patients

100ng of purified protein and 1:100 patient serum dilution was used in an indirect ELISA to estimate serum IgA, IgG and IgG-subclass response. Reactivity index (RI) values were calculated as a ratio between sample optical density and pre-pandemic negative samples. Each graph compares healthy individuals (n=13) and RT-PCR positive COVID-19 patients (n=128, samples n=211). Horizontal red line denotes the median antibody range.

Supplementary Figure 3: IgG4 response in COVID-19 patients

100ng of purified protein and 1:100 patient serum dilution was used in an indirect ELISA to estimate serum IgA, IgG and IgG-subclass response. Reactivity index (RI) values were calculated as a ratio between COVID-19 positive sample optical density and pre-pandemic negative samples. (A) Full-length and truncated SARS-CoV-2 nucleocapsid and spike proteins antibody binding is compared among COVID-19 patients (n=128). (B) Antibody reactivity is compared as a function of days after onset of symptoms. (C) Outpatients (n=80) and inpatients (n=48) or (D) discharged (n=24, samples n=59) and deceased (n=24, samples n=72) inpatients are compared. (E) IgG4 antibody response is compared between outpatients and inpatients =<7 days or >7 days post infection. ANOVA test with Tukey's post hoc test was used to compare differences in the RI between different antigen groups. Two-way ANOVA with Šidák post hoc correction for multiple comparisons. *$p < 0.05$, **$p < 0.01$, ***$p < 0.001$, ****$p < 0.0001$.

Supplementary Figure 4: Robust humoral response is detected one week post SARS-CoV-2 infection.

100ng of purified full-length and truncated SARS-CoV-2 proteins were coated on ELISA plates and 1:100 patient serum dilution was used in an indirect ELISA to estimate serum IgA, IgG and IgG-subclass response. RT-PCR confirmed COVID-19 patient's outpatients (n=80) and inpatients (n=48, samples n=131) were compared <=7 days or >7 days are the onset of symptoms. Horizontal red line denotes the median antibody range. Two-way ANOVA with Šidák post hoc correction for multiple comparisons. *$p < 0.05$, **$p < 0.01$, ***$p < 0.001$, ****$p < 0.0001$.

Supplementary Figure 5: Comparison of full-length nucleocapsid and truncated N2 protein reactivity among COVID-19 patients.
(A) Outpatient (n=5) and (B) inpatient (n=9) plasma samples were serially diluted and tested against 100ng of full-length nucleocapsid (N) or N2 protein in an indirect ELISA. Y-axis shows the optical density (OD) measured for these test samples against IgA or total IgG. Each coloured line represents one patient.

Supplementary Figure 6: Correlation between RBD-ACE2 inhibitory antibodies and SARS-CoV-2 antigen-specific response.
Inhibitory antibody levels were correlated with IgA, IgG and IgG-subclass reactivity against the full-length and truncated SARS-CoV-2 proteins. Each graph compares outpatient (n=70) and inpatient (n=41, samples n=120) with correlation and 95% CI. Inset depicts R squared values obtained by Spearman correlation.

Supplementary Figure 7: Correlation between neutralizing antibodies and SARS-CoV-2 antigen-specific response.
PRNT assay results were and correlated with IgA, IgG and IgG-subclass reactivity against the full-length and truncated SARS-CoV-2 proteins. Each graph compares all COVID-19 patients (n=54, samples n=82) with correlation and 95% CI. Inset depicts R squared values obtained by Spearman correlation.

Supplementary Figure 8: PRNT assay
Plasma samples were tested by PRNT in Vero cells after incubation with 300-plaque forming units (PFU). Percentage neutralization PRNT50 and PRNT90 represents the sample dilution corresponding 50% and 90% reduction in plaque formation compared to a control well inoculated with SARS-CoV-2 (without plasma). (A) Percentage neutralization was correlated with ACE2-RBD inhibitory antibody response among COVID-19 patient (n=54, samples n=82). (B) Outpatient (n=40) and inpatient (n=14) patient samples were serially diluted for the PRNT assay. Each data point represents the mean of all plasma samples for each group at each dilution level and error bars represent SD. (C and D) PRNT50 and PRNT90 values are compared to ACE2-RBD inhibitory antibody response. (E and F) days after onset of symptoms was compared to the PRNT50 and PRNT90 values for different study groups (outpatients n=40; discharged inpatients n=6, samples n=18; deceased inpatients n=8, samples n=24). Solid lines representing the tendency for the neutralizing antibodies was created by Fit Spline program GraphPad Prism software.

Supplementary Figure 9: Longitudinal humoral response dynamics in hospitalized patients.

100ng of purified full-length and truncated SARS-CoV-2 proteins were coated on ELISA plates and 1:100 patient serum dilution was used in an indirect ELISA to estimate serum IgA, IgG and IgG-subclass response. (A) RT-PCR confirmed hospitalized inpatient humoral response was compared in plasma samples collected longitudinally after hospital admission. Each line represents one patient and its antibody binding trajectories. Green colour denoted discharged inpatients (n=24) and pink denotes deceased inpatients (n=24). Solid line in black represents the tendency for the inpatients created by Fit Spline program GraphPad Prism software. X-axis denotes the RI and Y-axis days after onset of symptoms.

Supplementary Figure 10: Robust humoral response in severe patients despite low peripheral lymphocyte counts.
Lymphocyte data obtained by whole blood cell count was correlated with (A) anti-SARS-CoV-2 IgA or IgG response among COVID-19 patients (n=125, samples n=176) or (B) potentially neutralizing antibodies that block ACE2-RBD interactions. Each dot represents one patient sample. Lymphocyte levels are compared between (C) outpatients (n=78) and inpatients (n=47, samples n=98) or (D) discharged (n=23, samples n=40) and deceased (n=24, samples n=58) inpatients with blocking antibodies or days after onset of symptoms.

Supplementary Figure 11: Correlation matrix for study variables
A correlation analysis was performed for all variables described in the figure. Scale denotes spearman r value for each variable compared.

Supplementary Figure 12: Summary of serological response among COVID-19 patients.
(A) A principal component analysis was performed using all the available antibody reactivity variables, demographic, and clinical characteristics of the patients. (B) Coloured heat-map depicts relationship between patient characteristics, N3 and S1 binding antibodies and ACE2-RBD inhibitory antibodies.

Supplementary Figure 13: Longitudinal comparison of IgG subclass antibody response among COVID-19 patients.
Ratio of full-length and truncated N and S protein specific IgG subclass antibody response was compared days after onset of symptoms between outpatients (n=79) and inpatients (n=48, samples n=131) or hospitalized patients with discharged (n=24, samples n=59) or deceased (n=24, samples n=72) disease outcome. Solid lines represent the tendency for the antibody response created by Fit Spline program GraphPad Prism software.

Figure S1

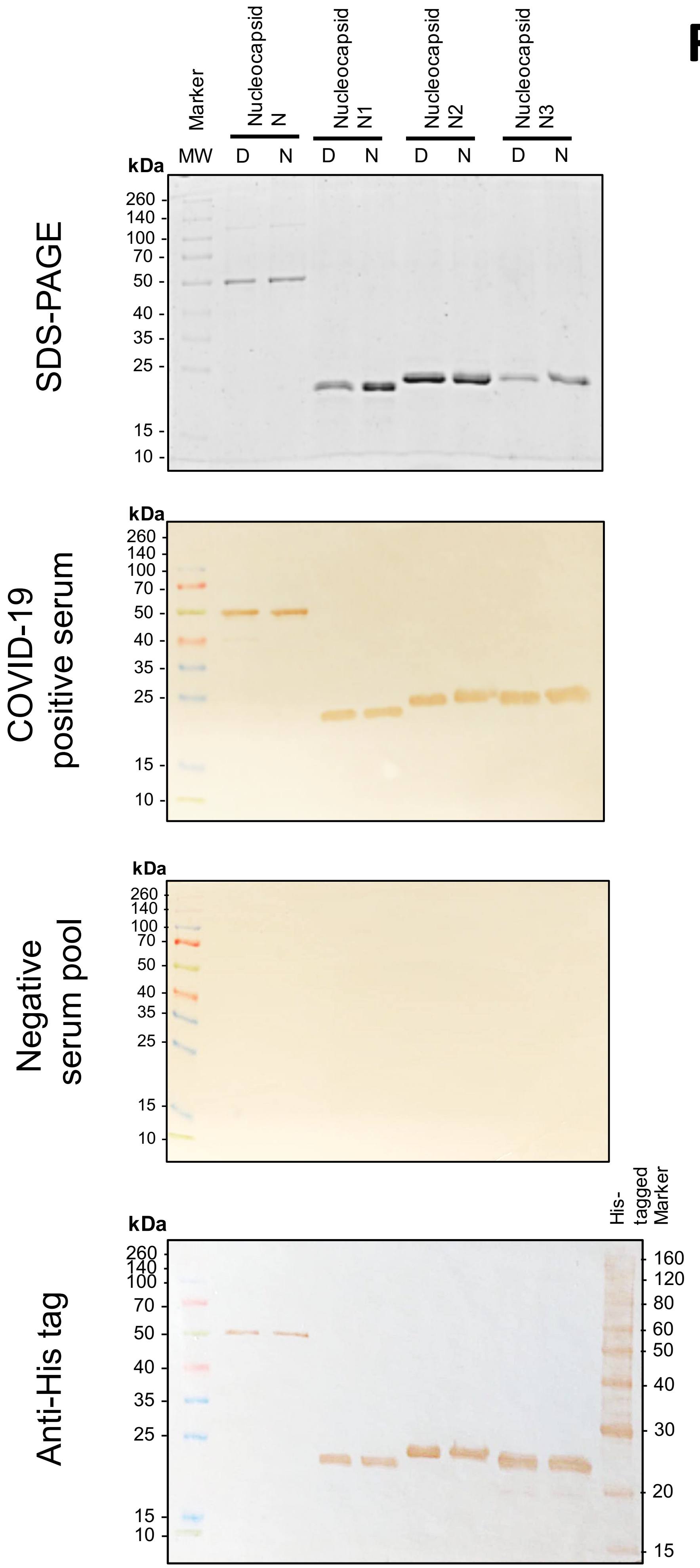

D: denatured protein
N: native protein

Figure S2

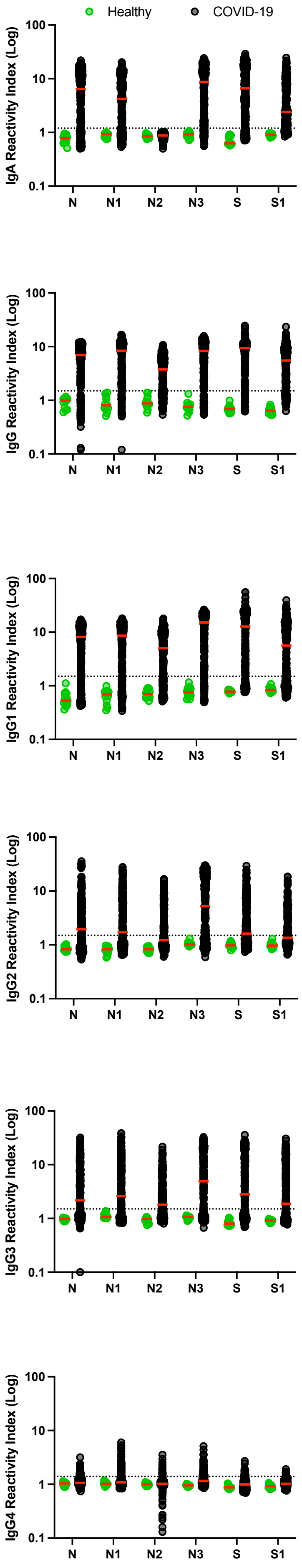
Healthy
COVID-19
IgA Reactivity Index (Log)
IgG Reactivity Index (Log)
IgG1 Reactivity Index (Log)
IgG2 Reactivity Index (Log)
IgG3 Reactivity Index (Log)
IgG4 Reactivity Index (Log)
100
10
1
0.1
N
N1
N2
N3
S
S1

Figure S3

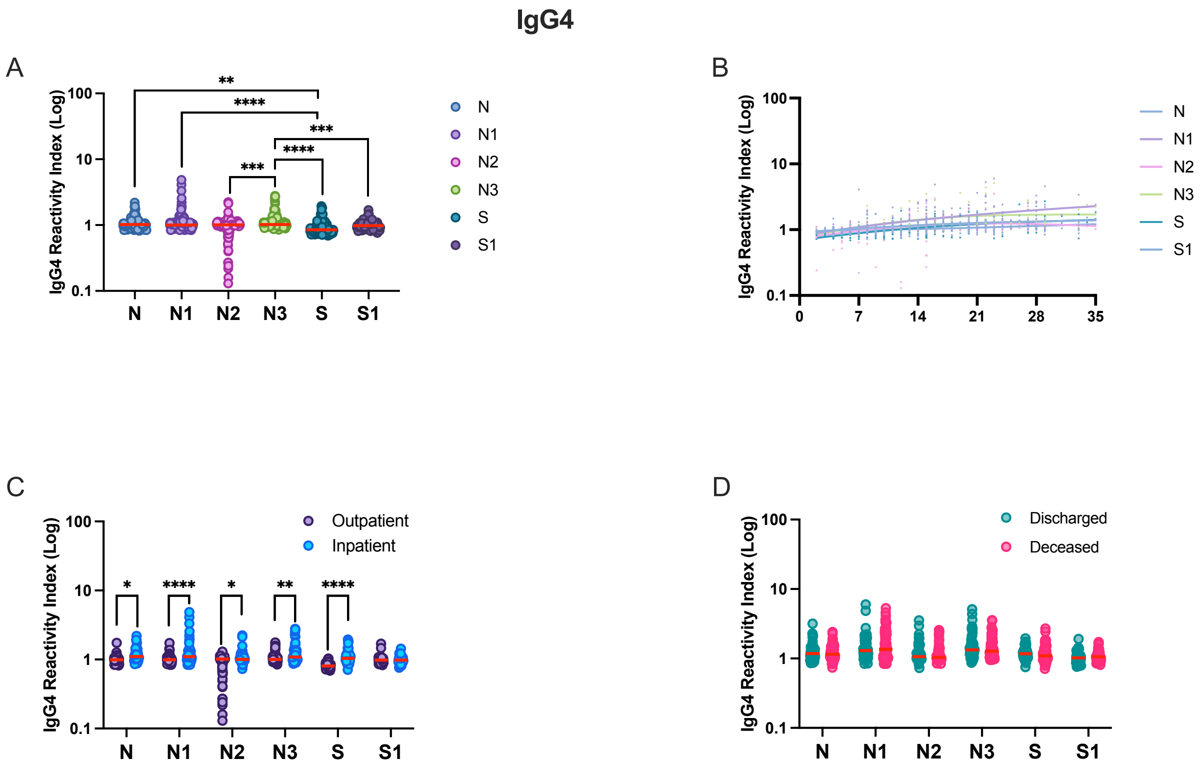
IgG4
A
IgG4 Reactivity Index (Log)
100
10
1
0.1
N
N1
N2
N3
S
S1
**
****
***
****
***
B
IgG4 Reactivity Index (Log)
0
7
14
21
28
35
C
IgG4 Reactivity Index (Log)
Outpatient
Inpatient
*
****
*
**
****
D
IgG4 Reactivity Index (Log)
Discharged
Deceased

Figure S4

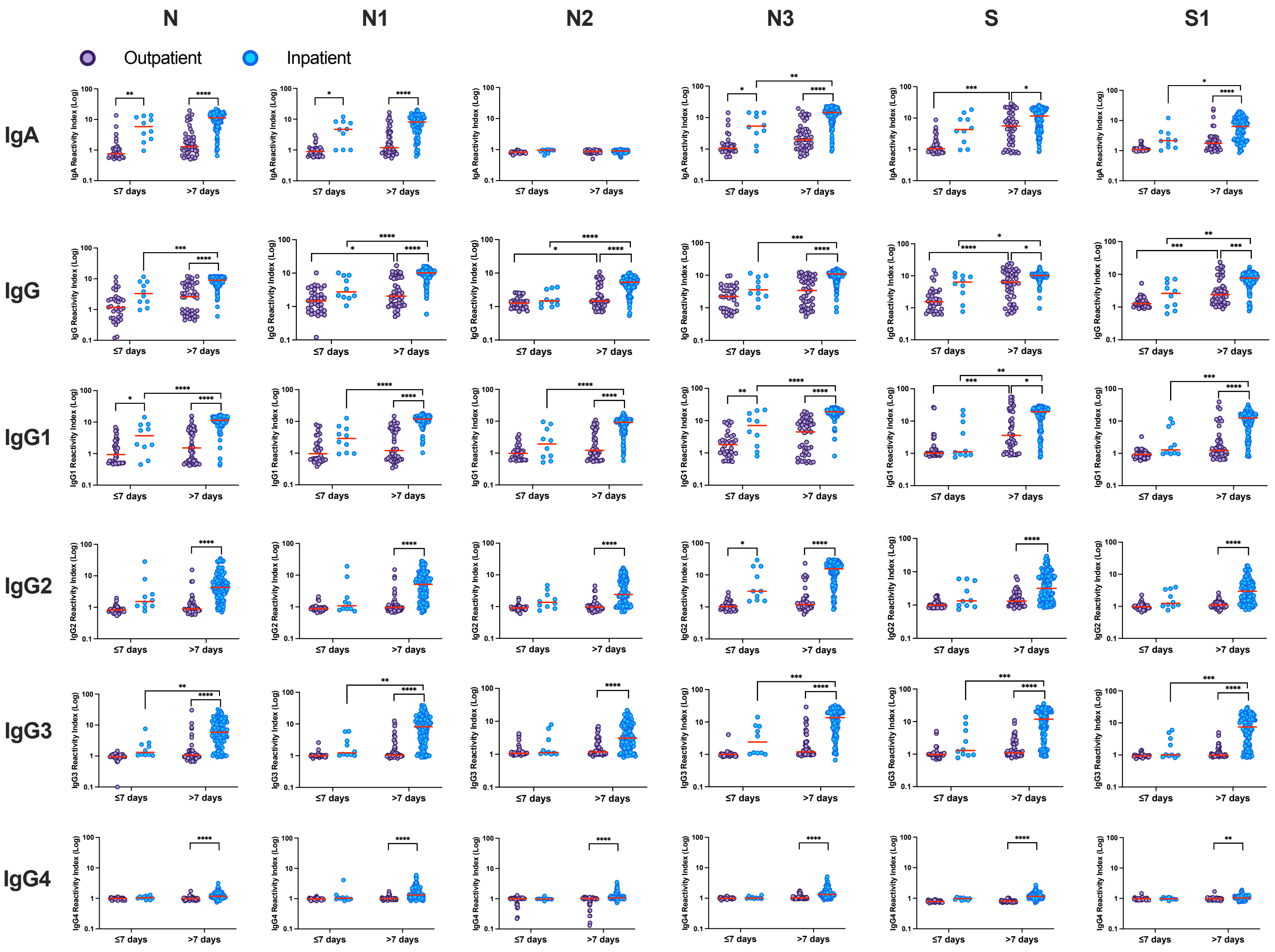
N
N1
N2
N3
S
S1
Outpatient
Inpatient
IgA
IgG
IgG1
IgG2
IgG3
IgG4
≤7 days
>7 days

Figure S5

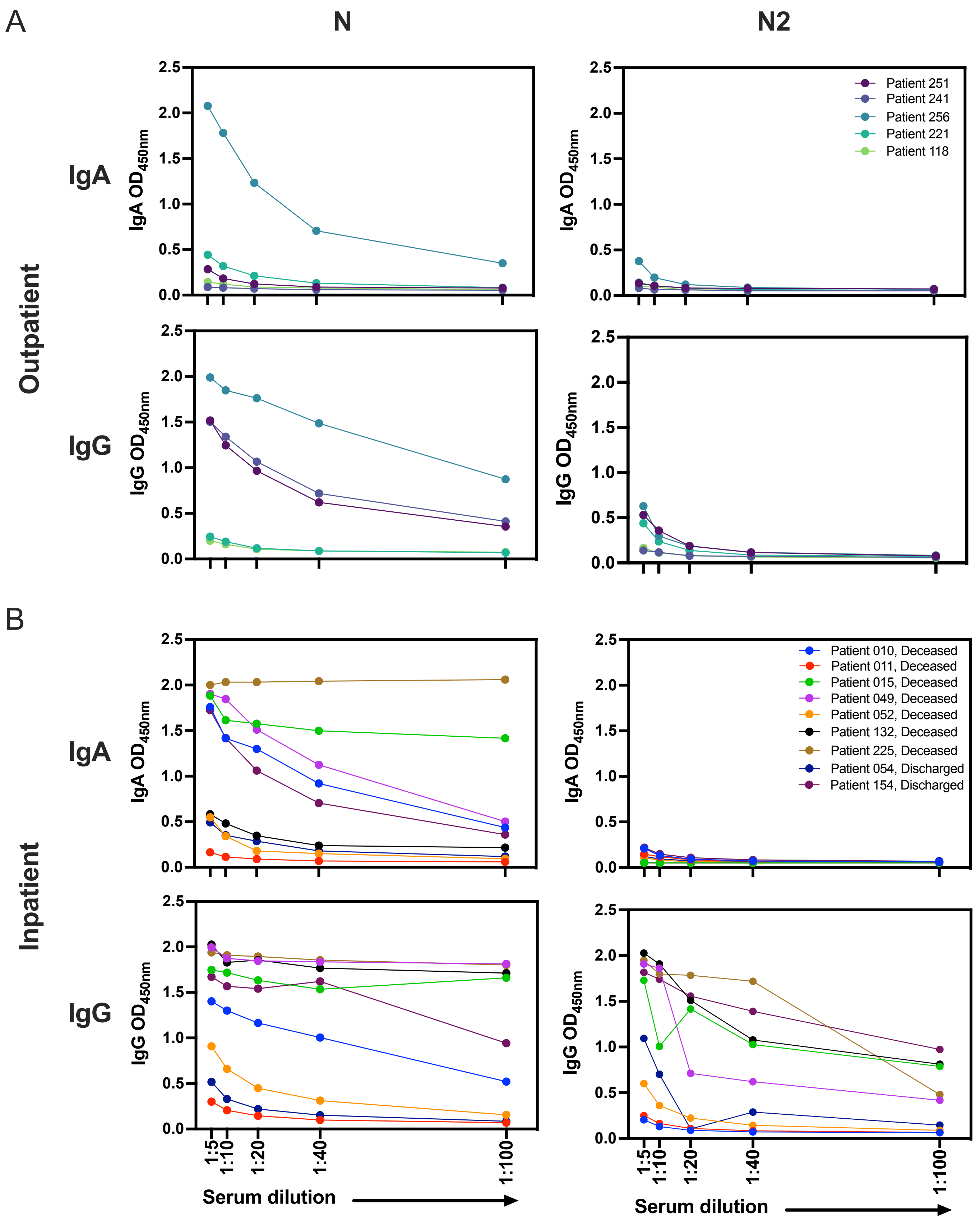
A
N
N2
Outpatient
IgA
IgG
IgA OD450nm
IgG OD450nm
Patient 251
Patient 241
Patient 256
Patient 221
Patient 118
B
Inpatient
Patient 010, Deceased
Patient 011, Deceased
Patient 015, Deceased
Patient 049, Deceased
Patient 052, Deceased
Patient 132, Deceased
Patient 225, Deceased
Patient 054, Discharged
Patient 154, Discharged
1:5
1:10
1:20
1:40
1:100
Serum dilution

Figure S6

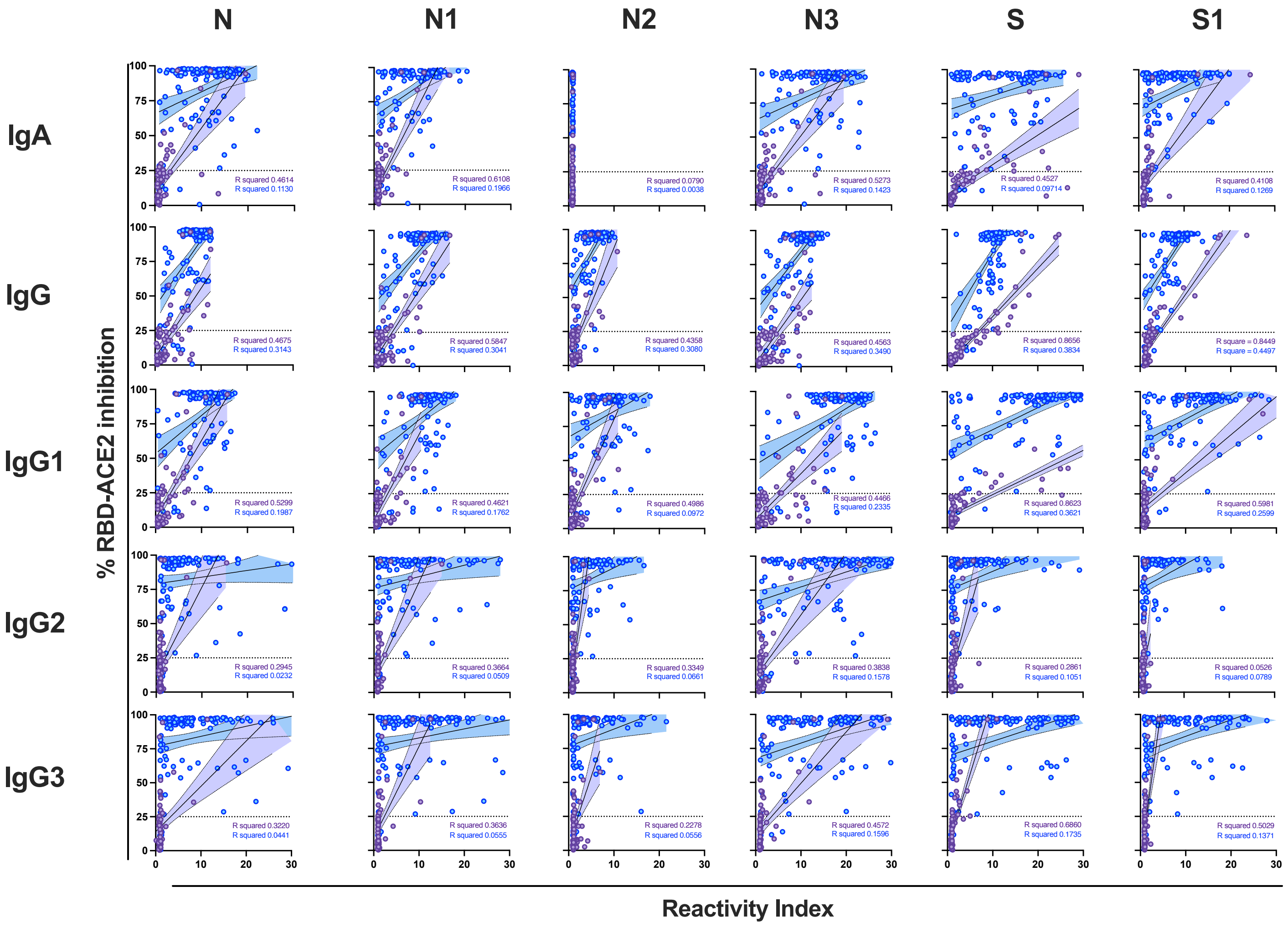
N
N1
N2
N3
S
S1
IgA
IgG
IgG1
IgG2
IgG3
% RBD-ACE2 inhibition
Reactivity Index
R squared 0.4614
R squared 0.1130
R squared 0.6108
R squared 0.1966
R squared 0.0790
R squared 0.0038
R squared 0.5273
R squared 0.1423
R squared 0.4527
R squared 0.09714
R squared 0.4108
R squared 0.1269
R squared 0.4675
R squared 0.3143
R squared 0.5847
R squared 0.3041
R squared 0.4358
R squared 0.3080
R squared 0.4563
R squared 0.3490
R squared 0.8656
R squared 0.3834
R square = 0.8449
R square = 0.4497
R squared 0.5299
R squared 0.1987
R squared 0.4621
R squared 0.1762
R squared 0.4986
R squared 0.0972
R squared 0.4466
R squared 0.2335
R squared 0.8623
R squared 0.3621
R squared 0.5981
R squared 0.2599
R squared 0.2945
R squared 0.0232
R squared 0.3664
R squared 0.0509
R squared 0.3349
R squared 0.0661
R squared 0.3838
R squared 0.1578
R squared 0.2861
R squared 0.1051
R squared 0.0526
R squared 0.0789
R squared 0.3220
R squared 0.0441
R squared 0.3636
R squared 0.0555
R squared 0.2278
R squared 0.0556
R squared 0.4572
R squared 0.1596
R squared 0.6860
R squared 0.1735
R squared 0.5029
R squared 0.1371

Figure S7

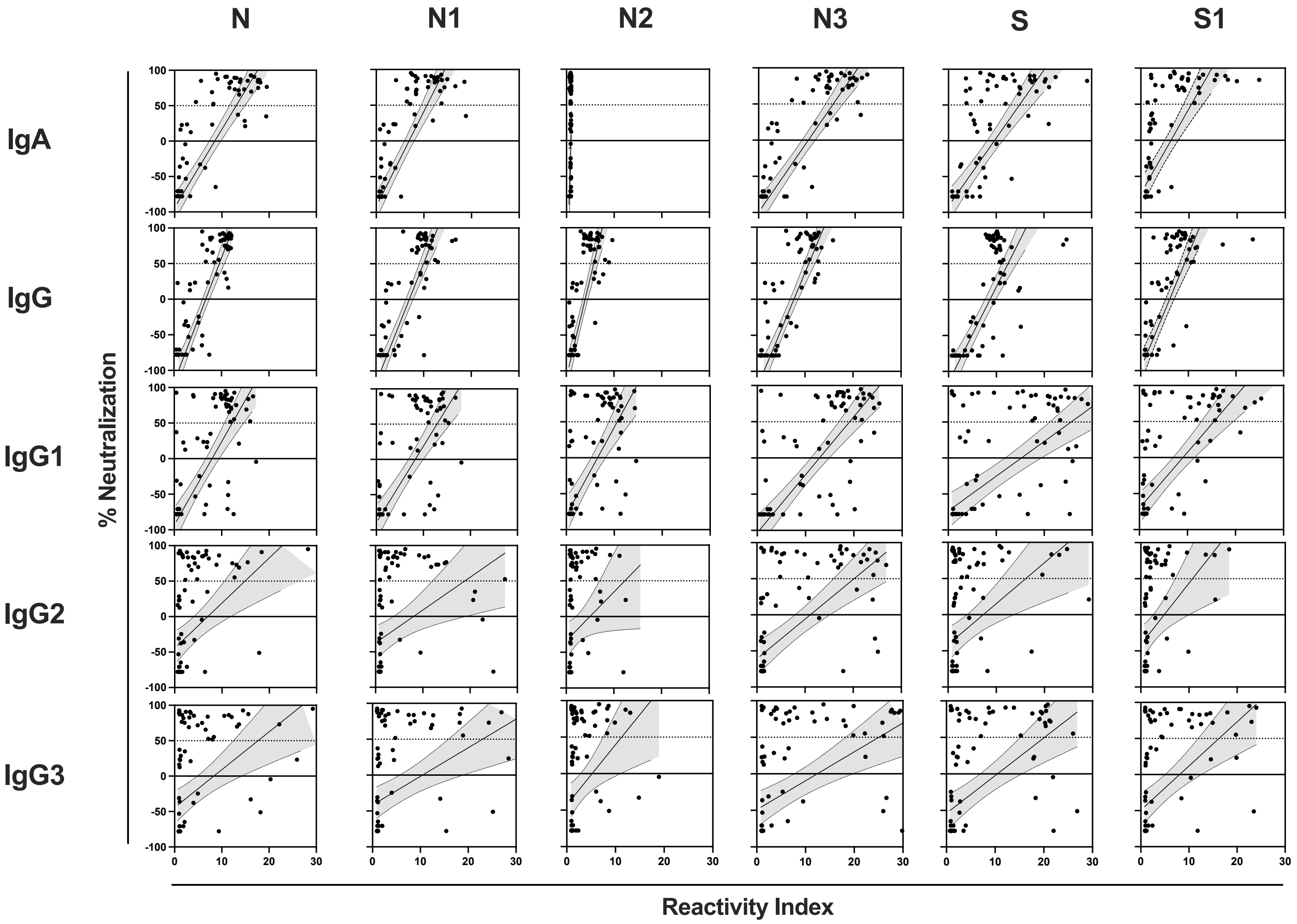
N
N1
N2
N3
S
S1
IgA
IgG
IgG1
IgG2
IgG3
% Neutralization
Reactivity Index

Figure S8

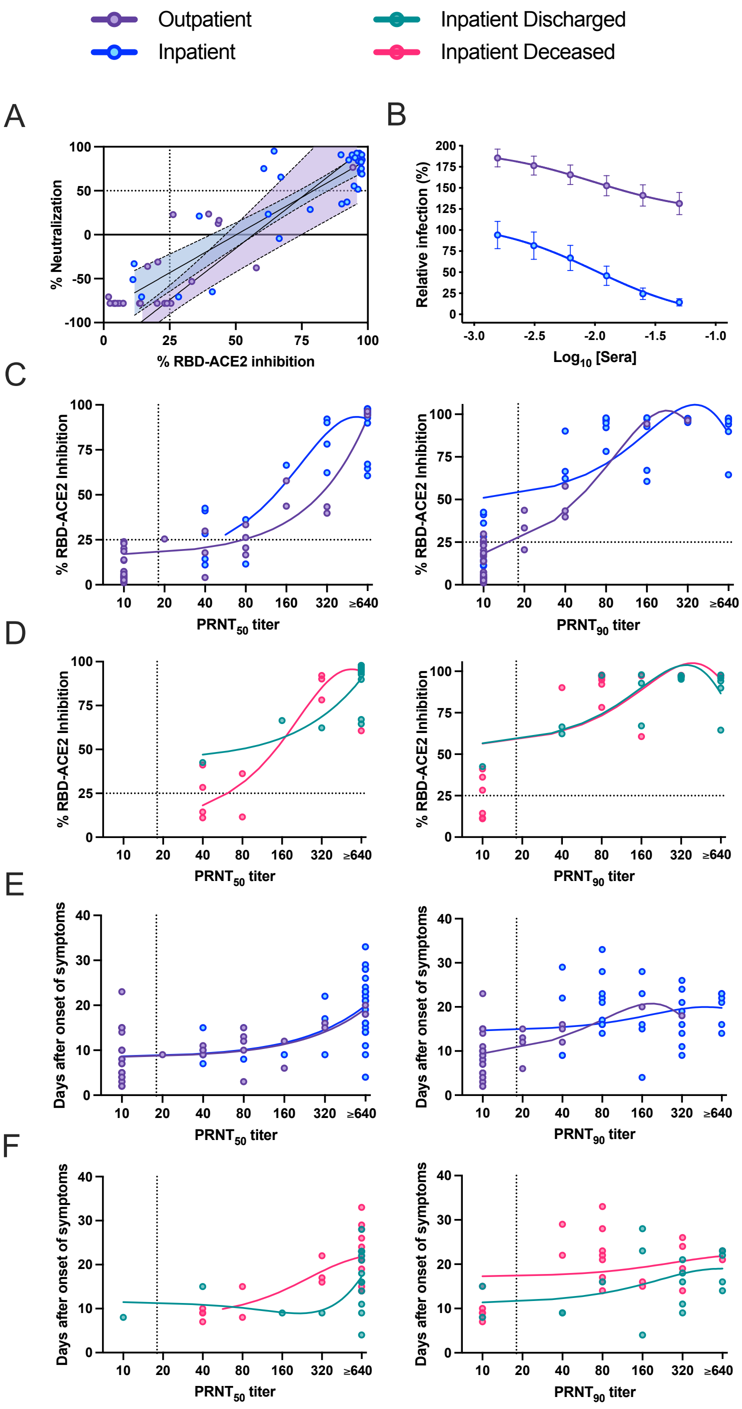

Outpatient
Inpatient
Inpatient Discharged
Inpatient Deceased
A
% Neutralization
% RBD-ACE2 inhibition
B
Relative infection (%)
Log10 [Sera]
C
% RBD-ACE2 Inhibition
PRNT50 titer
PRNT90 titer
D
% RBD-ACE2 Inhibition
PRNT50 titer
PRNT90 titer
E
Days after onset of symptoms
PRNT50 titer
PRNT90 titer
F
Days after onset of symptoms
PRNT50 titer
PRNT90 titer

Figure S9

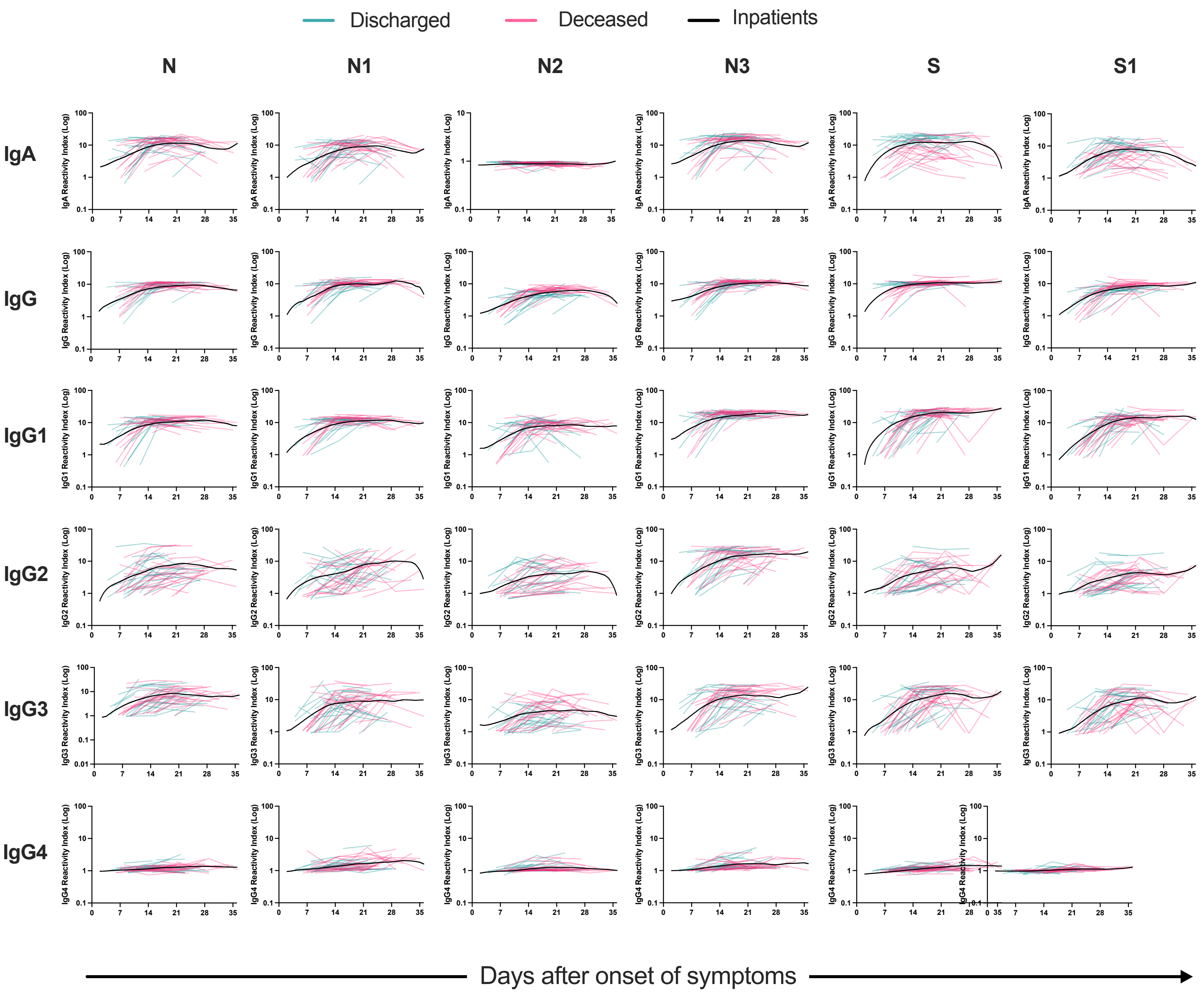

Discharged
Deceased
Inpatients
N
N1
N2
N3
S
S1
IgA
IgG
IgG1
IgG2
IgG3
IgG4
Days after onset of symptoms

Figure S10

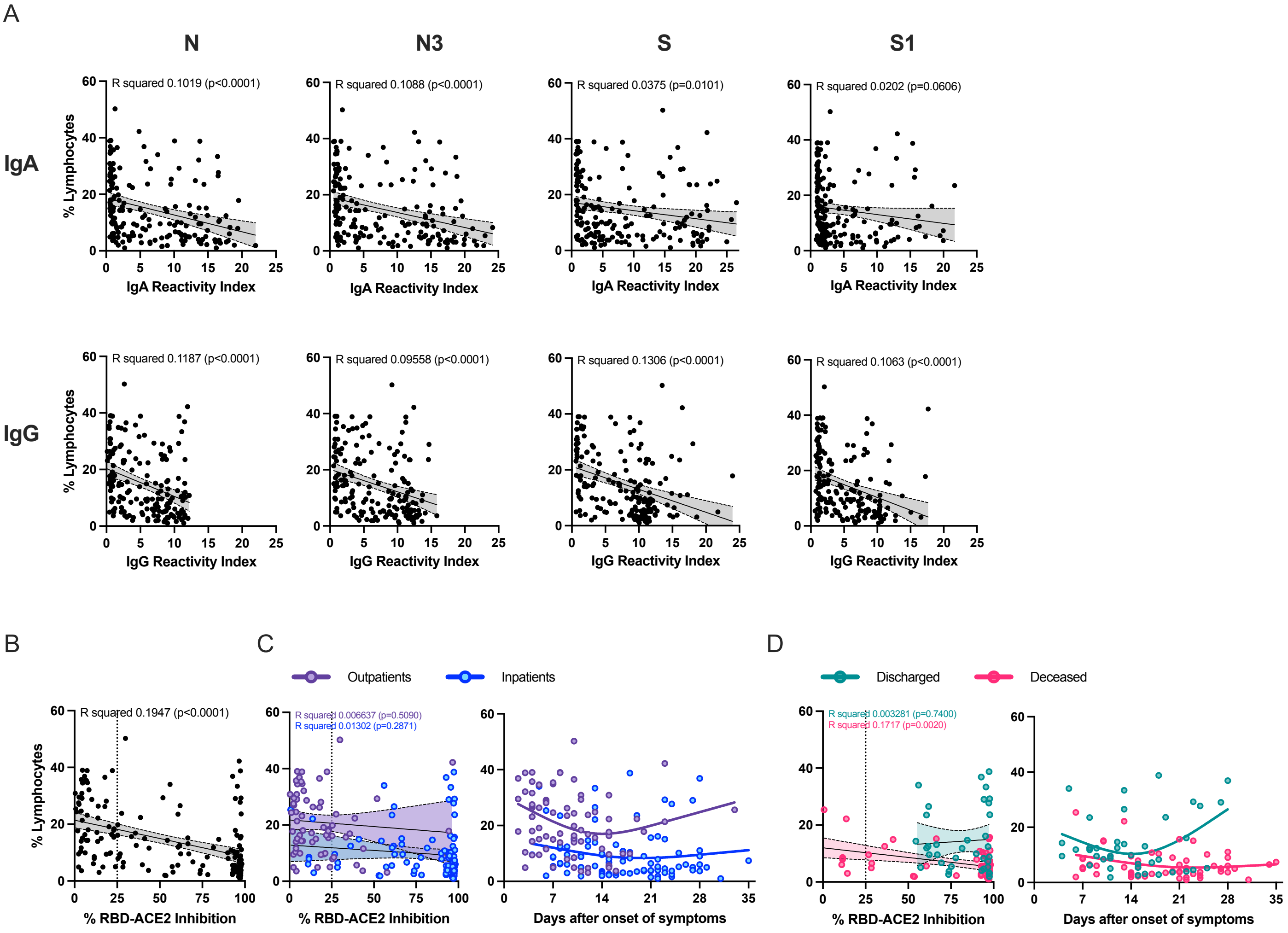
A
N
N3
S
S1
IgA
IgG
% Lymphocytes
R squared 0.1019 (p<0.0001)
R squared 0.1088 (p<0.0001)
R squared 0.0375 (p=0.0101)
R squared 0.0202 (p=0.0606)
IgA Reactivity Index
R squared 0.1187 (p<0.0001)
R squared 0.09558 (p<0.0001)
R squared 0.1306 (p<0.0001)
R squared 0.1063 (p<0.0001)
IgG Reactivity Index
B
C
D
R squared 0.1947 (p<0.0001)
% RBD-ACE2 Inhibition
Outpatients
Inpatients
R squared 0.006637 (p=0.5090)
R squared 0.01302 (p=0.2871)
Days after onset of symptoms
Discharged
Deceased
R squared 0.003281 (p=0.7400)
R squared 0.1717 (p=0.0020)

Figure S11

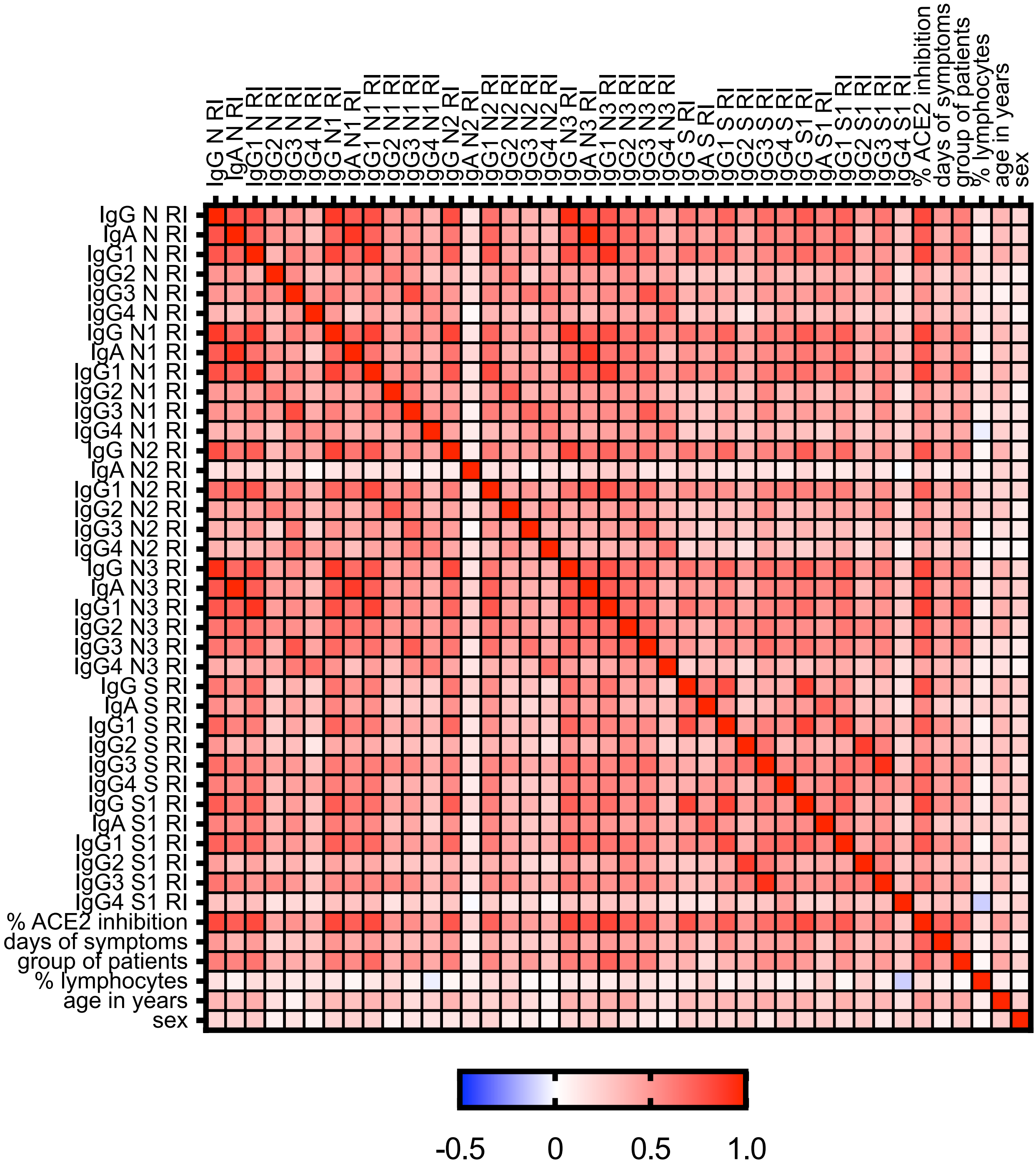
IgG N RI
IgA N RI
IgG1 N RI
IgG2 N RI
IgG3 N RI
IgG4 N RI
IgG N1 RI
IgA N1 RI
IgG1 N1 RI
IgG2 N1 RI
IgG3 N1 RI
IgG4 N1 RI
IgG N2 RI
IgA N2 RI
IgG1 N2 RI
IgG2 N2 RI
IgG3 N2 RI
IgG4 N2 RI
IgG N3 RI
IgA N3 RI
IgG1 N3 RI
IgG2 N3 RI
IgG3 N3 RI
IgG4 N3 RI
IgG S RI
IgA S RI
IgG1 S RI
IgG2 S RI
IgG3 S RI
IgG4 S RI
IgG S1 RI
IgA S1 RI
IgG1 S1 RI
IgG2 S1 RI
IgG3 S1 RI
IgG4 S1 RI
% ACE2 inhibition
days of symptoms
group of patients
% lymphocytes
age in years
sex
-0.5
0
0.5
1.0

Figure S12

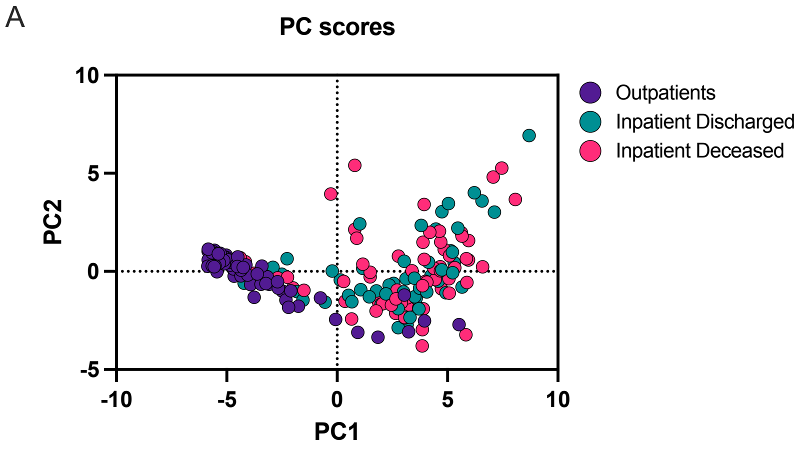

A
PC scores
PC2
PC1
10
5
0
-5
-10
-5
0
5
10
Outpatients
Inpatient Discharged
Inpatient Deceased

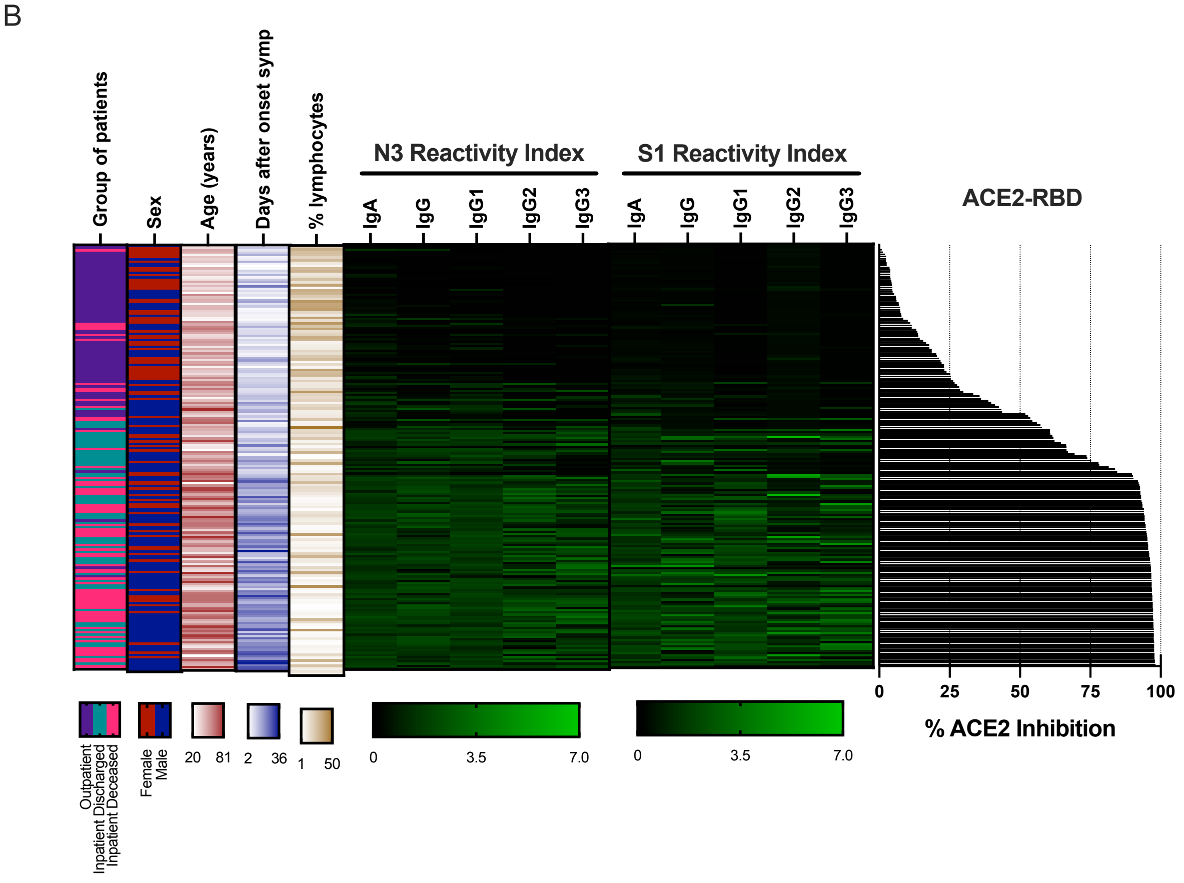

B
Group of patients
Sex
Age (years)
Days after onset symp
% lymphocytes
N3 Reactivity Index
S1 Reactivity Index
IgA
IgG
IgG1
IgG2
IgG3
ACE2-RBD
0
25
50
75
100
% ACE2 Inhibition
Outpatient
Inpatient Discharged
Inpatient Deceased
Female
Male
20
81
2
36
1
50
0
3.5
7.0

Figure S13

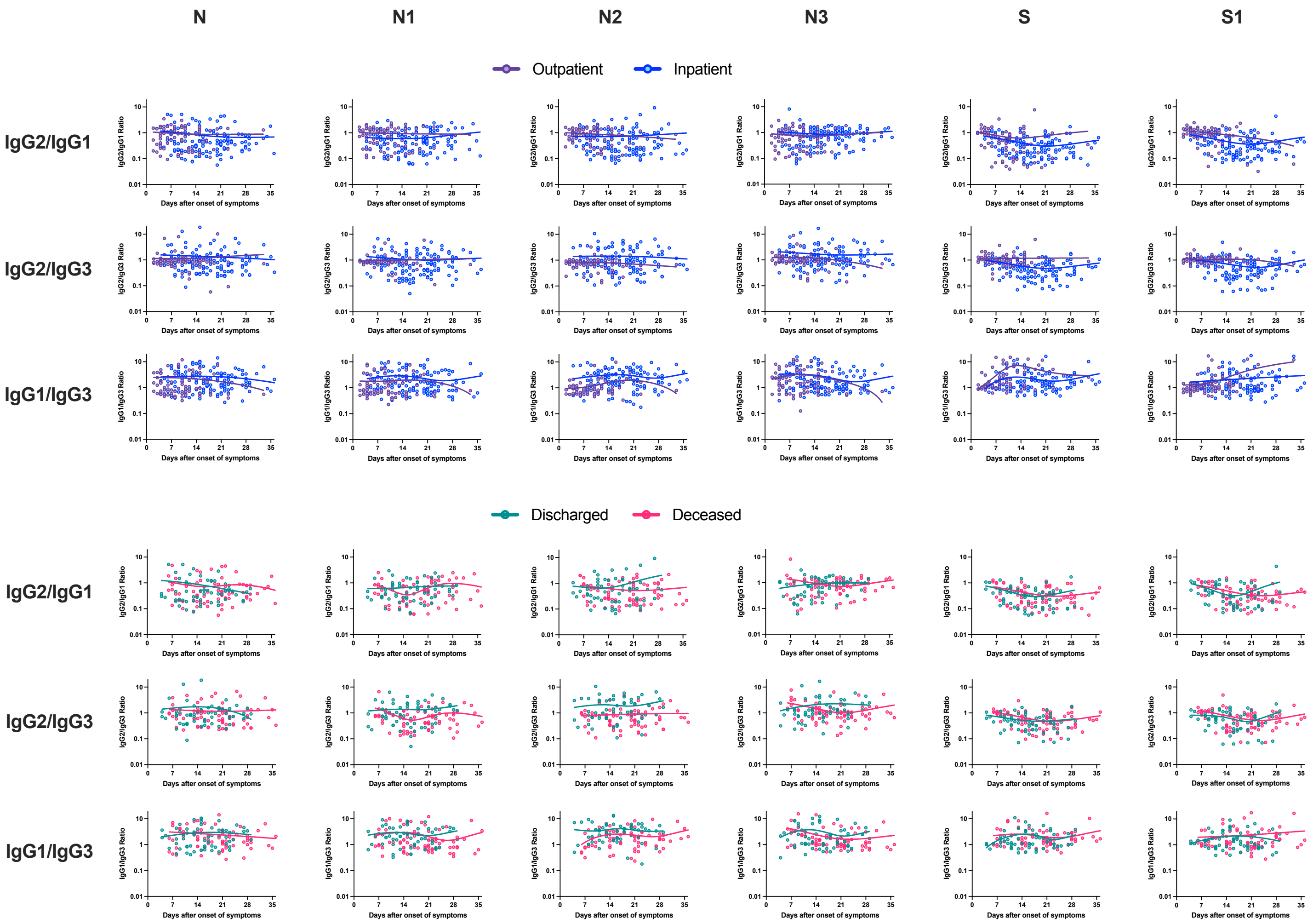

N
N1
N2
N3
S
S1
Outpatient
Inpatient
IgG2/IgG1
IgG2/IgG3
IgG1/IgG3
Discharged
Deceased
IgG2/IgG1 Ratio
IgG2/IgG3 Ratio
IgG1/IgG3 Ratio
10
1
0.1
0.01
0
7
14
21
28
35
Days after onset of symptoms
