# Supplementary material for: Antigen-Specific Antibody Signature Is Associated with COVID-19 Outcome
Source: Viruses. 2023 Apr 20;15(4):1018. doi: 10.3390/v15041018 (PMC10143282; doi:10.3390/v15041018)
Supplement: Supplementary file 1 [file viruses-15-01018-s001.zip › viruses-2142507-supplementary/viruses-2142507-Supplementary Materials/Salgado et.al_Supplementary Tables.pdf]

Supplementary Table 1. Demographic, clinical and hematological characteristics of COVID-19 patients.

Characteristics		Outpatients n=80	Inpatients n=48	P value	Inpatients Discharged n=24	Inpatients Deceased n=24	P value
Age median (IQR)		40.5 (33 - 47)	55.5 (45 - 63)		52.5 (41.25 – 62.75)	58.5 (47.0 – 64.0)	
range		21 - 64	20 - 81		27 - 81	20 – 79	
Gender	Male	36 (45)	34 (70.8)		17 (70.8)	17 (70.8)	
	Female	44 (55)	14 (29.2)		7 (29.2)	7 (29.2)	
Race	Brown	63 (78.7)	37 (77.1)		18 (75.0)	19 (79.2)	
	Black	4 (5.0)	5 (10.4)		3 (12.5)	2 (8.3)	
	White	9 (11.3)	6 (12.5)		3 (12.5)	3 (12.5)	
	East Asian	4 (5.0)	0		0	0	
BMI median (IQR)		29.2 (25.9 - 33.1)	29.25 (25.9 - 32.4)		29.25 (25.75 – 32.38)	29.70 (26.05 – 35.00)	
BMI range (minimum- maximum)		17.9 - 49.9	22.8 - 58.8		22.6 – 58.8	23.3 – 43.4	
Symptoms Days median (IQR)		9 (5 - 33)	10.5 (8 - 22)		10.5 (8.0 – 13.0)	10.5 (7.25 – 15.0)	
Comorbidities*	Chronic heart disease	3/80 (3.75)	6/48 (12.5)	0.0789	3/24 (12.5)	3/24 (12.5)	
	Hypertension	23/80 (28.75)	21/48 (43.75)	0.5876	8/24 (33.3)	13/24 (54.2)	
	Pulmonary disease	4/80 (5.0)	4/48 (8.3)	0.4721	3/24 (12.5)	1/24 (4.17)	
	Tuberculosis	2/80 (2.5)	1/48 (2.1)	0.9999	1/24 (4.17)	0/24 (0)	
	Diabetes	12/80 (15.0)	14/48 (29.16)	0.0698	8/24 (33.3)	6/24 (25.0)	
	Obesity	36/80 (45.0)	21/48 (43.75)	0.9999	10/24 (41.7)	11/24 (45.8)	
	Alcoholism	48/80 (60.0)	20/48 (41.67)	0.8525	10/24 (41.7)	10/24 (41.7)	
	HIV/AIDS	1/80 (1.25)	0/48 (0)	0.9999	0/24 (0)	0/24 (0)	
	Kidney disease	5/80 (6.25)	0/48 (0)	0.1562	0/24 (0)	0/24 (0)	
	Neurological disease	8/80 (10.0)	3/48 (6.25)	0.5345	1/24 (4.17)	2/24 (8.3)	
	Rheumatic disease	5/80 (6.25)	5/48 (10.42)	0.5	5/24 (20.8)	0/24 (0)	
	Smoking habit	21/80 (26.25)	14/48 (29.16)	0.8384	6/24 (25.0)	8/24 (33.3)	
	Liver disease	3/80 (3.75)	0/48 (0)	0.2913	0/24 (0)	0/24 (0)	
	Chronic hematological disease	4/80 (5.0)	0/48 (0)	0.2692	0/24 (0)	0/24 (0)	
Clinical symptoms*	Fever	73/80 (91.25)	43/48 (89.6)	0.7623	22/24 (91.6)	21/24 (87.5)	
	Dry cough	71/80 (88.75)	42/48 (87.5)	0.9999	21/24 (87.5)	21/24 (87.5)	
	Sore throat	51/80 (63.75)	22/48 (45.8)	0.0649	12/24 (50.0)	10/24 (41.6)	
	Nasal congestion	61/80 (76.25)	18/48 (37.5)	0.0001	10/24 (41.6)	8/24 (33.3)	
	Nasal discharge	49/80 (61.25)	19/48 (39.6)	0.0276	12/24 (50.0)	7/24 (29.17)	
	Earache	28/80 (35.0)	7/48 (14.6)	0.014	6/24 (25.0)	1/24 (4.17)	
	Wheezing	31/80 (38.75)	17/48 (35.4)	0.8506	5/24 (20.8)	12/24 (50.0)	
	Chest Pain	62/80 (77.5)	31/48 (64.6)	0.1513	16/24 (66.7)	15/24 (62.5)	
	Myalgia	72/80 (90.0)	30/48 (62.5)	0.0005	18/24 (75.0)	12/24 (50.0)	
	Arthralgia	64/80 (80.0)	24/48 (50.0)	0.0007	13/24 (54.17)	11/24 (45.8)	
	Fatigue	75/80 (93.75)	42/48 (87.5)	0.3285	21/24 (87.5)	21/24 (87.5)	
	Shortness of breathe	70/80 (87.5)	45/48 (93.7)	0.3683	22/24 (91.6)	23/24 (95.8)	
	Intercostal circulation	41/80 (51.25)	20/48 (41.6)	0.3614	12/24 (50.0)	8/24 (33.3)	
	Nasal Wing Beating	23/80 (28.75)	12/48 (25.0)	0.687	8/24 (33.3)	4/24 (16.67)	
	Anosmia	66/80 (82.5)	28/48 (58.3)	0.0001	15/24 (62.5)	13/24 (54.17)	
	Dysgeusia (loss of taste)	66/80 (82.5)	23/48 (47.9)	0.0001	15/24 (62.5)	8/24 (33.3)	

	Dehydration	44/80 (55.0)	14/48 (29.2)	0.0058	7/24 (29.17)	7/24 (29.17)	
	Headache	72/80 (90.0)	35/48 (72.9)	0.0146	21/24 (87.5)	14/24 (58.3)	
	Chill	60/80 (75.0)	26/48 (54.2)	0.0197	15/24 (62.5)	11/24 (45.8)	
	Dizziness	51/80 (63.75)	22/48 (45.8)	0.0649	15/24 (62.5)	7/24 (29.17)	
	Convulsions	4/80 (5.0)	4/48 (8.3)	0.4721	2/24 (8.33)	2/24 (8.33)	
	Altered Consciousness	19/80 (23.7)	5/48 (10.4)	0.0665	2/24 (8.33)	3/24 (12.5)	
	Abdominal pain	42/80 (52.5)	23/48 (47.9)	0.7154	14/24 (58.3)	9/24 (37.5)	
	Vomiting	15/80 (18.7)	14/48 (29.2)	0.195	7/24 (29.17)	7/24 (29.17)	
	Nausea	52/80 (65.0)	20/48 (41.6)	0.0163	11/24 (45.8)	9/24 (37.5)	
	Diarrhea	41/80 (51.2)	22/48 (45.8)	0.5876	13/24 (54.17)	9/24 (37.5)	
	Conjunctivitis	3/80 (3.75)	4/48 (8.3)	0.4237	1/24 (4.17)	3/24 (12.5)	
	Rash	8/80 (10.0)	1/48 (2.1)	0.1519	1/24 (4.17)	0/24 (0)	
	Lymphadenopathy	22/80 (27.5)	4/48 (8.3)	0.1978	0/24 (0)	4/24 (16.67)	
	Sputum	37/80 (46.2)	27/48 (56.2)	0.3614	13/24 (54.17)	14/24 (58.3)	
	Itching	11/80 (13.7)	1/48 (2.1)	0.0305	1/24 (4.17)	0/24 (0)	
	Skin Injuries	3/80 (3.75)	0/48 (0)	0.2913	0/24 (0)	0/24 (0)	
	Bleeding (Hemorrhage)	1/80 (1.25)	2/48 (4.2)	0.5557	1/24 (4.17)	1/24 (4.17)	
Blood count#, mean (95% CI)	Hemoglobin (g/dL)	13.44 (12.95 – 13.94)	12.69 (12.13 – 13.25)	0.053	13.17 (12.50 – 13.85)	12.22 (11.33 – 13.11)	0.087
	Leukocytes (mm^3)	8.39 (7.54 – 9.24)	11.79 (9.93 – 13.64)	0.0002	10.62 (8.39 – 12.85)	12.91 (9.86 – 15.94)	0.2184
	Lymphocytes (%)	21.17 (18.69 – 23.64)	10.15 (8.08 – 12.23)	<0.0001	11.93 (8.67 – 15.19)	8.45 (5.79 – 11.11)	0.092
	Neutrophils (%)	70.61 (67.49 – 73.72)	81.75 (77.87 – 85.63)	<0.0001	81.28 (77.39 – 85.17)	82.20 (75.25 – 89.16)	0.8146
	Hematocrit (%)	46.87 (43.77 – 49.97)	39.51 (38.01 – 41.00)	0.0006	40.72 (38.96 – 42.49)	38.34 (35.92 – 40.77)	0.1099
	Platelets ($10^3/mm^3$)	259.94 (240.2 – 279.7)	301.04 (267.7 – 334.4)	0.025	301.7 (257.6 – 345.7)	300.5 (247.1 – 353.8)	0.9718
	ALT (U/L)	52.61 (44.30 – 60.91)	85.5 (62.34 – 108.70)	0.001	97.64 (62.39 – 132.9)	69.32 (38.91 – 99.73)	0.2207
	AST (U/L)	43.74 (37.76 – 49.71)	87.46 (57.49)	<0.0001	77.16 (54.78 – 99.55)	101.2 (31.88 – 170.5)	0.4260
	Glucose (mg/dL)	173.14 (110.1 – 236.2)	208.58 (173.7 – 243.4)	0.3775	214.4 (151.6 – 277.1)	203.4 (161.7 – 245.2)	0.7559
	Creatinine (mg/dL)	3.6 (-1.74 – 8.94)	1.57 (1.15 – 1.99)	0.5719	1.22 (0.85 – 1.59)	1.90 (1.15 – 2.64)	0.1059
	Urea (mg/dL)	27.92 (26.10 – 29.74)	56.04 (41.63 – 70.45)	<0.0001	42.18 (28.33 – 56.03)	69.27 (44.47 – 94.06)	0.0569
	LDH (U/L)	441.32 (144.8 – 737.9)	972.68 (619.9 – 1,326.0)	0.1573	796.7 (648.8 – 944.6)	1,149 (408.7 – 1,889)	0.3112
	C-reactive protein (mg/dL)	60.5 (0.1 – 120.9)	101.84 (76.87 – 126.8)	0.2028	94.20 (55.32 – 133.1)	109.50 (74.99 – 144.0)	0.5428
Hospitalization#	Symptoms days before hospitalization median (IQR)		8.0 (6.0 – 13.0)		8.0 (6.0 – 12.0)	8.5 (6.0 – 13.0)	0.3991
	Number of days in hospital median (IQR)		16.0 (13.0 – 23.75)		13.50 (11.0 – 23.0)	19.0 (14.25 – 24.50)	0.0711
	Symptoms days until outcome median (IQR)		28.0 (20.0 – 34.0)		24.0 (18.0 – 33.0)	29.50 (24.0 – 37.5)	0.0356
	ICU		29/48 (60.4)		5/24 (20.8)	24/24 (100)	<0.0001
	Number of days in ICU median (IQR)		16.0 (11.5 – 21.5)		19.0 (8.5 – 27.0)	16.0 (12.25 – 20.0)	0.9259
	Mechanical ventilation use*		28/48 (58.3)		5/24 (20.8)	23/24 (95.83)	<0.0001
	Number of days on ventilation median (IQR)		16.0 (12.0 – 20.0)		12.0 (5.5 – 20.5)	17.0 (14.0 – 20.0)	0.1191

The data from comorbidities and clinical symptoms are represented as number of individuals with the finding in question among total and the relative percentage as the proportion of these individuals in the group. #P value from Unpaired T test. *P value from Fisher's exact test. IQR: interquartile ranges; BMI: Body mass index; ALT: Alanine transaminase; AST: Aspartate transaminase; LDH: lactate dehydrogenase; ICU: Intensive Care Unit.

Supplementary Table 2. Summary of patients and tests performed.

Method	Group of patients	Number of patients	Number of samples
ELISA*<br>IgA<br>IgG<br>IgG1<br>IgG2<br>IgG3<br>IgG4	Healthy[#]	13	224
	Outpatients[§]	80	
	Inpatients Discharged[^]	24 (59[@])	
	Inpatients Deceased[^]	24 (72[@])	
NeutraLISA	Healthy[#]	13	203
	Outpatients[§]	70	
	Inpatients Discharged[^]	20 (54[@])	
	Inpatients Deceased[^]	21 (66[@])	
PRNT[&]	Outpatients[§]	40	82
	Inpatients Discharged[^]	6 (18[@])	
	Inpatients Deceased[^]	8 (24[@])	

*All antibodies detection assays were assessed for N, N1, N2, N3, S and S1 antigens;
[#]People without symptoms and SARS-CoV-2 negative;
[§]Symptomatics, with or without fever and SARS-CoV-2 positive;
[^]Symptoms as dyspnea, respiratory discomfort, O2 saturation < 94%, Severe Acute Respiratory Syndrome and SARS-CoV-2 positive.
[@]Total number of samples, including longitudinal samples of patients
[&]Each sample was tested in duplicate with 2-fold serial dilutions
